# Dual-Step Controlled Release of Berberine Hydrochloride from the Trans-Scale Hybrids of Nanofibers and Microparticles

**DOI:** 10.3390/biom13061011

**Published:** 2023-06-18

**Authors:** Jianfeng Zhou, Yelin Dai, Junhao Fu, Chao Yan, Deng-Guang Yu, Tao Yi

**Affiliations:** 1 School of Materials and Chemistry, University of Shanghai for Science and Technology, Shanghai 200093, China; 221550217@st.usst.edu.cn (J.Z.); 2035053109@st.usst.edu.cn (J.F.); 2026010226@st.usst.edu.cn (C.Y.); 2 Wenqi Middle School, East Jiangchuan Road 980, Shanghai 200240, China; daiyelin2007@163.com; 3 High School Affiliated to Fudan University, Qingpu Campus, Longpu Road 500, Shanghai 201700, China; 4 Faculty of Health Sciences and Sports, Macao Polytechnic University, Macau 999078, China

**Keywords:** dual-step release, berberine hydrochloride, hybrid, electrospinning, nanofibers, electrospraying, microparticle

## Abstract

In this nano era, nanomaterials and nanostructures are popular in developing novel functional materials. However, the combinations of materials at micro and macro scales can open new routes for developing novel trans-scale products with improved or even new functional performances. In this work, a brand-new hybrid, containing both nanofibers and microparticles, was fabricated using a sequential electrohydrodynamic atomization (EHDA) process. Firstly, the microparticles loaded with drug (berberine hydrochloride, BH) molecules in the cellulose acetate (CA) were fabricated using a solution electrospraying process. Later, these microparticles were suspended into a co-dissolved solution that contained BH and a hydrophilic polymer (polypyrrolidone, PVP) and were co-electrospun into the nanofiber/microparticle hybrids. The EHDA processes were recorded, and the resultant trans-scale products showed a typical hybrid topography, with microparticles distributed all over the nanofibers, which was demonstrated by SEM assessments. FTIR and XRD demonstrated that the components within the hybrids were presented in an amorphous state and had fine compatibility with each other. In vitro dissolution tests verified that the hybrids were able to provide the designed dual-step drug release profiles, a combination of the fast release step of BH from the hydrophilic PVP nanofibers through an erosion mechanism and the sustained release step of BH from the insoluble CA microparticles via a typical Fickian diffusion mechanism. The present protocols pave a new way for developing trans-scale functional materials.

## 1. Introduction

As an advanced drug controlled release profile, dual-step release (or biphasic release) has its advantages of endowing a fast therapeutic action effect and a long time period of constant blood drug concentration for the patients’ conveniences [1,2,3,4,5,6,7]. In general, the first phase fast or pulsatile release of a drug is able to promote the drug concentration in the blood to a therapeutic window. Later, the second phase release is realized through a sustained or extended manner, by which the drug blood concentration is kept within the therapeutic window [8,9,10,11,12,13,14,15]. Thus, their combination, on one hand, can accelerate the therapeutic action. On the other hand, it can reduce the oral administration times for an increased patients’ compliance [16,17,18,19].

Berberine hydrochloride (BH), a popular biomolecule with broad functional performances, is a typical poorly water-soluble drug. It can be extracted from coptis chinensis and belongs to a chemical drug purified from traditional Chinese medicine. BH has a broad antibacterial spectrum and has antibacterial effects on a variety of Gram-positive and negative bacteria, including *hemolytic streptococci*, *Staphylococcus aureus*, *Vibrio cholera*, *Meningococci*, *Shigella dysentery bacilli*, *Typhoid bacilli*, and *Diphtheria bacilli* [20]. At a low concentration, it inhibits bacteria, while at a high concentration, it kills bacteria. BH also has certain inhibitory effects on influenza viruses, amoeba, leptospira, and certain skin fungi [21]. In vitro experiments confirmed that BH can enhance the phagocytic ability of white blood cells and the hepatic reticuloendothelial system. BH has a therapeutic effect on helicobacter, and can alleviate gastritis, gastric and duodenal ulcers. It is reported that BH can reduce the number of pili on the surface of the bacterial body, preventing bacteria from adhering to human cells and, thus, has the therapeutic functional performance [22]. BH has no cross resistance to penicillin, streptomycin, etc. However, the oral absorption of BH is very poor. Furthermore, it quickly enters various organs and tissues and distributed widely (mostly in the heart, bone, lung, and liver) after intramuscular injection, and the blood drug concentration is maintained to the level over the minimum inhibitory concentration for only a very short time period [23]. Thus, a frequent oral administration of the commercial BH products (such as tablets, capsules, and pills) is needed for the patients. Biphasic release of BH may benefit an improved therapeutic effect after oral administration.

During the past several decades, although many new excipients (including organic materials such as polymers and lipids, inorganic materials, their composites and hybrids [24,25,26,27,28,29]) and new strategies were frequently introduced into the pharmaceutical field for endowing the active ingredients a better therapeutic effect [30,31,32,33,34], the mainstream is still the traditional pharmaceutical excipients. This is because these excipients were demonstrated to be safe and compatible with organisms. Thus, for a certain drug, it is the material conversion methods that played their important roles in endowing an improved functional application. Particularly in this nano era, nano fabrication methods are continuously adopted by pharmacists to convert the drug molecules and excipient molecules to medicated nanoproducts for realizing the designed therapeutic effects [35,36]. Numerous examples can be found in the literature. One example is the electrospun nanofibers of traditional hydrophilic polymeric excipients (e.g., PVA, PVP, PEO, gelatin, and so on) for fast dissolution and therapeutic action of a poorly water-soluble drug [37,38,39,40,41]. Another example is the electrosprayed microparticles and electrospun nanofibers of conventional insoluble or biodegradable polymeric excipients (e.g., CA, EC, PLA, PCL, PAN, and zein) for a designed drug sustained release profile [42,43,44,45,46,47].

Based on the hints from the above-mentioned studies, the present work investigated a combination of electrospinning and electrospraying (both belong to the technique of electrohydrodynamic atomization, EHDA) [48,49,50], by which a new type of hybrids consisting of both electrospun nanofibers and electrosprayed microparticles were fabricated for providing a biphasic release of BH. The prepared hybrids were subjected to a series of characterizations including their morphology, the physical state and compatibility of the loaded components, and the in vitro drug controlled release profiles. Both the EHDA mechanism and drug biphasic release mechanism are proposed.

## 2. Materials and Methods

### 2.1. Materials

Berberine hydrochloride (BH, purity over 98%) was purchased from a local Laobaixing Drugstore (Shanghai, China). Cellulose acetate (Mw = 30,000) was bought from Aldrich. Polyvinylpyrrolidone K90 (Mw = 1,300,000) was purchased from Sigma-Aldrich Corp. (Shanghai, China) The solvents acetone, ethanol, Di-ChloroMethane (DCM) and N, N-Dimethylacetamide (DMAc) were obtained from Shanghai Fitst Shiji Factory (Shanghai, China). Water was double distilled just before use.

### 2.2. Fabrication Methods

Two different EHDA processes (i.e., a single-fluid blending electrospraying and a single-fluid blending electrospinning) were arranged in a sequential manner for preparing the hybrids of electrospun nanofibers and electrosprayed microparticles.

According to literature [51], CA is soluble in a mixture of acetone/ethanol/DMAc with a volume ratio of 4:1:1. Meanwhile, the drug BH is soluble in DMAc; thus, a co-dissolving working fluid (Fluid 1, Table 1) containing CA and BH could be prepared for the single-fluid electrospraying process. After some pre-experiments, 15 g BH and 25 g CA were co-dissolved in 500 mL of the solvent mixture.

A homemade EHDA apparatus was exploited to conduct the electrospraying process. The experimental conditions include an applied voltage of 20 kV, a fluid flow rate of 1.0 mL/h, and a particle deposition distance of 20 cm from the nozzle of spinneret to the grounded collector. The environmental temperature and humidity were 21 ± 5 °C and 47 ± 7%, respectively.

An amount of 15.0 g microparticles E1 from electrospraying was suspended into 200 mL Fluid 2 (containing 36.0 g PVP and 4.0 g BH in 400 mL mixture of DCM and DMAc with a volume ratio of 9:1) uniformly through continuous stirring to form a suspension working fluid (Fluid 3, Table 1). During the electrospinning process, little sedimentation of the microparticles was observed, which should be attributed to the relatively lower density of electrosprayed microparticles.

### 2.3. Characterization

#### 2.3.1. Morphology

The morphologies of the EHDA products were evaluated using a field-emission scanning electron microscope (SEM, Quanta FEG450, Hillsboro, OR, USA). The SEM pictures were used to estimate the average diameters of the nanofibers in about 100 places using the ImageJ software (National Institutes of Health, Bethesda, MD, USA). The sampling processes included fixing some powders E1 or a strip of E2 and E3 on a sample holder using a double-sided conductive adhesive, and a thin layer of Au was sprayed for 60 s before assessments under an applied voltage of 5.0 kV.

#### 2.3.2. Physical State and Compatibility among the Components

X-ray diffraction (XRD) tests were carried out using the Bruker X-ray Powder diffractometer (Bruker-AXS, Karlsruhe, Germany). The raw materials and their fiber mats were measured within a 2θ angle range of 5°–60°. The applied voltage and working current were 40 kV and 30 mA, respectively. The rotation speed was 5° per minute.

Fourier transform infrared (FTIR) analyses were implemented using a PerkinElmer FTIR Spectrometer (Spectrum 100, Billerica, MA, USA). The experiments were performed in range 500–4000 cm^−1^ with a resolution of 2 cm^−1^. The sampling for the solid materials included weighing 0.2 g of potassium bromide powder, grinding it with about 10 mg of the sample, pressing the mixture into solid tablets, and placing the tablets into the instrument for scanning.

#### 2.3.3. Functional Performances

The drug release profiles of the three EHDA products were assessed using the paddle method in accordance with the Chinese Pharmacopoeia (2020 Ed.). Approximately 200 mg of the EHDA products were placed into a vessel with 900 mL phosphate-buffered solution (PBS, 0.1 M, pH = 7.0). The dissolution media were maintained at 37 ± 1 °C and a rotation rate of 50 rpm. At predetermined time points, a volume of 5.0 mL aliquot was withdrawn and filtered through a 0.22 μm membrane (Millipore, MA, USA). Five milliliters of fresh PBS was added to maintain a constant dissolution bulk volume. The amounts of BH released were measured at λ_max_ = 263 nm using a UV-vis spectrophotometer (UV-2102PC, Unico Instrument Co. Ltd., Shanghai, China). A calibration equation was pre-determined for calculating the BH concentration. The experimental results were reported as mean ± S.D. All experiments were repeated six times.

## 3. Results and Discussion

### 3.1. The Sequential EHDA Process

**Figure 1:**
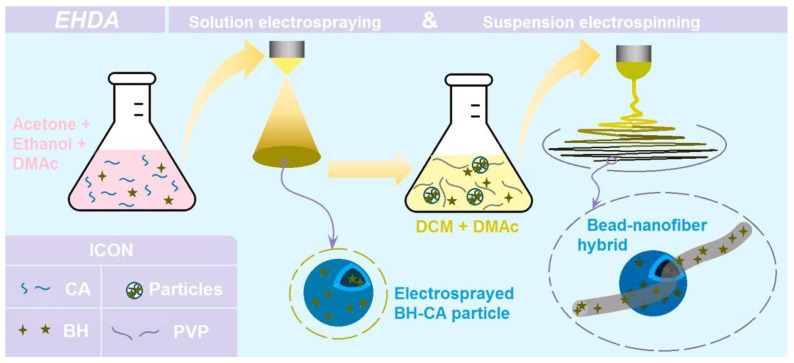
A diagram showing the fabrication procedures of the hybrids composed of the electrospun nanofibers and the electrosprayed microparticles.

EHDA processes are hydrodynamic atomization procedures that are initiated by an applied high voltage, and they are exploited to prepare solid products by taking advantages of the easy interactions between electrostatic energy and working fluids [52,53]. For electrospinning, the solid products are often nanofibers resulted from the continuous drawing of viscous polymer fluid jets [54,55,56]. For electrospraying, the solid products are typically microparticles that resulted from the fission and repelling of droplets [57]. Thus, based on the capabilities of these two EHDA processes, a new type of trans-scale hybrids can be conceived as Figure 1, and three kinds of EHDA products were fabricated according to the conditions listed in Table 1.

Firstly, the water-insoluble polymer CA and drug BH were co-dissolved into a solvent mixture containing acetone, ethanol, and DMAc with a volume ratio of 4:1:1. After some pre-experiments, the solid microparticles could be prepared through a single-step and straightforward electrospraying process. Later, these microparticles were dispersed into the co-dissolving solution containing PVP and BH in a solvent mixture containing DCM and DMAc with a volume ratio of 9:1. The CA particles did not dissolve in the solvent mixture. Thus, the working fluid was a suspension. After the electrospinning of the suspension, the hybrids containing both nanofibers of PVP and microparticles of CA were prepared, and the drug BH was distributed both in the nanofibers and also in the microparticles.

**Figure 2:**
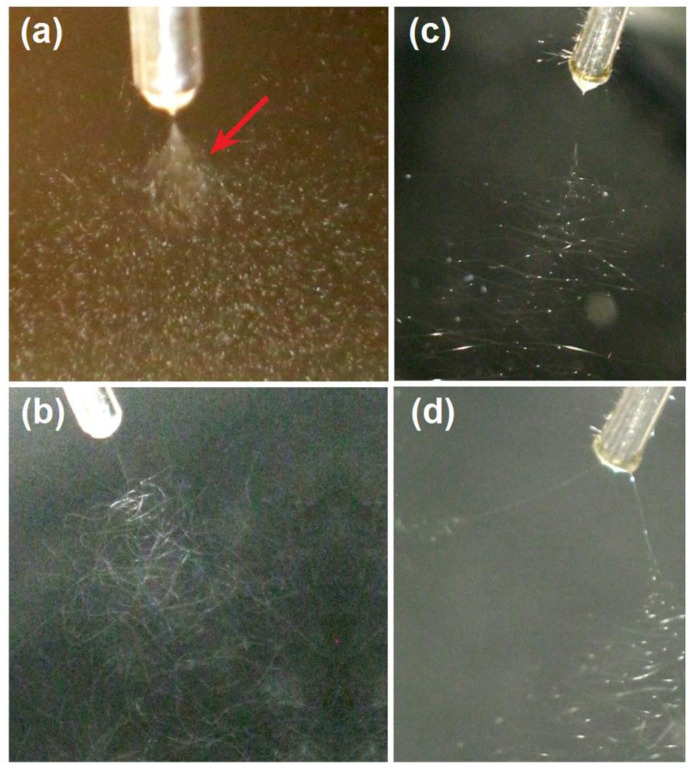
Digital pictures taken from the different EHDA working processes: (**a**) a typical electrospraying process for fabricating the microparticles E1, the red arrow indicates a splitted and fast moving region; (**b**) a typical solution electrospinning process for creating the nanofibers E2; (**c**) a typical suspension electrospinning process for producing hybrids of E3; (**d**) an abnormal EHDA process when treating the suspension under a super high applied voltage.

For a successful electrospinning process, the working fluid must be electrospinnable [58,59]. Thus, a relatively high polymer concentration is needed to keep enough physical entanglements of polymeric molecules in the working fluid, by which the electrostatic drawing can be resisted for elongating the fluid jets till the formation of nanofibers [60,61,62]. However, for a successful electrospraying process, the working fluid needs only to be solidifiable, i.e., the effective removement of organic solvents [63]. Thus, the polymer concentration is often lower than the electrospinnable one. Showed in Figure 2a is the typical digital picture taken from the electrospraying processes for fabricating the microparticles E1. At the top of Taylor cone, an opposite cone can be observed for the Coulombic expansion. The “white” section region, indicated by a red arrow, was formed by the fast moving speed of the fast splitted droplets. As the droplets splitted and reduced their sizes and weights, their movings were decelerated, which can be recorded by a digital camera (30 frames per second). The PVP-BH solution had fine electrospinnability; a typical working process is given in Figure 2b, by which the composite nanofibers E2 were fabricated. Figure 2c exhibits the typical suspension electrospinning process for producing hybrids E3.

The comparison between Figure 2b,c can tell the influence of the added CA microparticles on the electrospinning processes. Although under the same experimental conditions (a fixed applied voltage, flow rate, and a collected distance of 12 kV, 2.0 mL/h, and 20 cm, respectively), the three sections (Taylor cone, straight fluid jet, and bending and whipping region [64,65,66,67]) had significant differences. The Taylor cone and straight fluid jet of the treated suspension had a larger volume and a longer size than those of treated PVP solution. Apparently, the “large” weight of microparticles played their role in enlarging the Taylor cone and elongating the straight fluid jet. Furthermore, in the bending and whipping process of the unstable regions, it was clear that the bright dots, formed by the microparticles, were always there from the end of straight fluid jet to the deposition just above the collector. Meanwhile, the suspension jets movings showed the unsmooth polygonal lines, different with the smooth and continuous lines of solution jets movement trajectory. Still, the microparticles clung on the jets resulted in these complex phenomena. During the electrospinning of suspension for fabricating hybrids E3, the fluid jets were more easily separated when a small elevation of the applied voltage to 15 kV. A typical record is shown in Figure 2d. After separation into two branches, the branch that contained more microparticles mainly moved downwards, owing to the presence of microparticles. Meanwhile, the easy aggregation of electric charges on the surface of microparticles should be also a reason for easy separation under a higher voltage. Thus, keeping a suitable applied voltage can benefit a higher quality of EHDA hybrids in terms of the uniform distribution of microparticles within the nanofibers. During all the processes, clogging of spinneret was seldom observed. In this work, two different kinds of EHDA processes were combined. Along this way, many other laboratory or even industrial techniques (such as supercritical technology and three-dimensional printing) can be combined with EHDA to develop novel materials conversion methods [68,69,70].

### 3.2. The Morphologies of the Resultant Products

**Figure 3:**
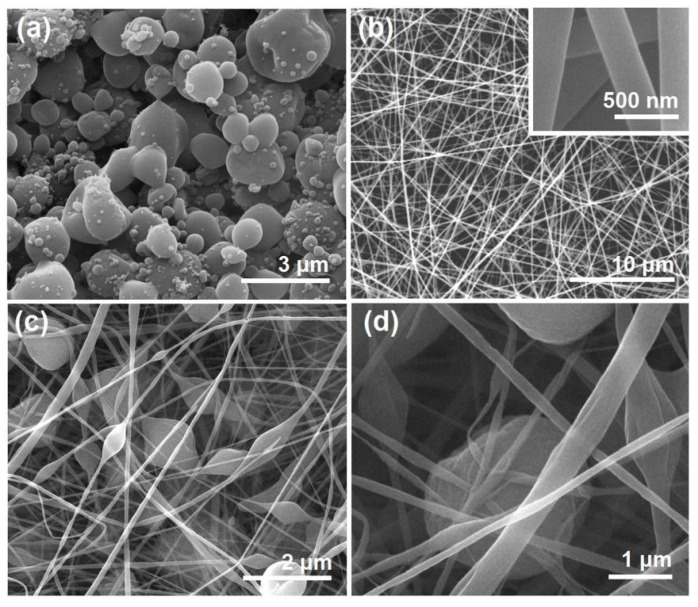
SEM images of the resultant products: (**a**) microparticles E1; (**b**) nanofibers E2, the up-right inset shows an enlarged image; (**c**) hybrids E3; (**d**) an enlarged image of hybrids E3.

The morphologies of the different EHDA products are included in Figure 3. Interestingly, there were many satellites around the electrosprayed microparticles E1 (Figure 3a). For the application of drug sustained release, these satellites may be a negative factor due to the extremely small size and correspondingly the short routes for the loaded drug molecules diffusion from them to the bulk solutions. Just as anticipated, the electrospun BH-PVP nanofibers E2 had fine linear morphology without any discerned beads or spindles (Figure 3b). Meanwhile, an enlarged image in the up-right inset of Figure 3b indicates that these nanofibers had a very smooth surface without the possible drug particles formed by phase separation during the storage process. The electrospun hybrids from the suspensions exhibited a typical “mixture” of beads or spindles and nanofibers, as indicated by Figure 3c,d.

**Figure 4:**
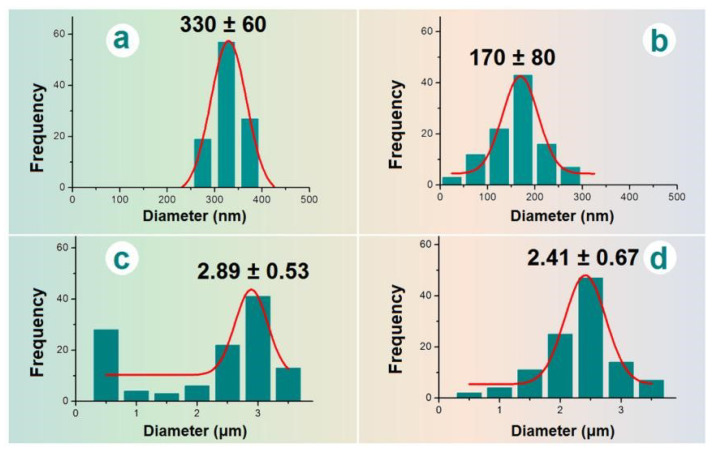
The average diameters of EHDA products: (**a**) nanofibers E2; (**b**) the nanofibers of hybrids E3; (**c**) microparticles E1; (**d**) the microparticles of hybrids E3.

The sizes and size distributions of these EHDA products were estimated using ImageJ software. All the results are shown in Figure 4. The average diameters of nanofibers E2 were 330 ± 60 nm (Figure 4a). In sharp contrast, the diameters of the nanofiber sections in the electrospun hybrids E3 were only 170 ± 80 nm (Figure 4b). The difference was a direct result of the added microparticles, whose presence should increase the drawing effects on the moving fluid jets and, in turn, a significant reduction in the nanofibers’ diameter. The electrosprayed microparticles E2 had an average diameter of 2.89 ± 0.53 μm (Figure 4c). In contrast, the diameter of particular sections in electrospun hybrids E3 was 2.41 ± 0.67 μm (Figure 4d), showing a slight reduction in the average size. This indicates that the re-dispersing of microparticles E1 in the solvent mixture of DCM and DMAc for preparing the suspension working fluid may result in a little influence on their final morphology and size. Although CA is insoluble in DCM and DMAc, the drug BH distributed on the surface of microparticles may re-dissolve into the suspensions and build a dynamic balance between the surfaces of microparticles and the bulk suspensions. 

**Figure 5:**
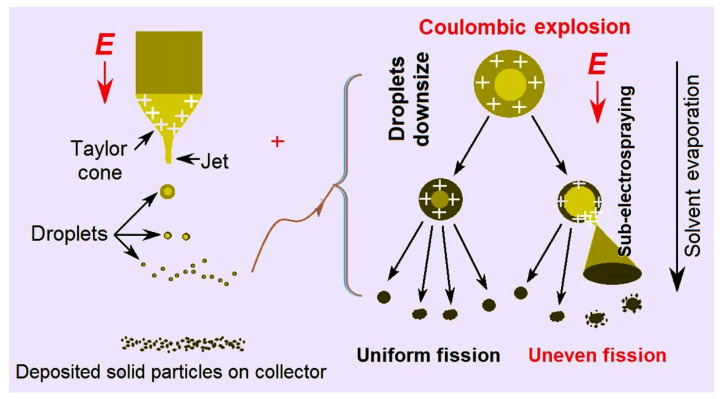
The EHDA mechanism for the formation of satellites around the electrosprayed microparticles.

Although there is no general theory for instructing the implementation of EHDA processes, there are abundant suggested mechanisms in the literature for the treatment of a certain working fluid, both for electrospinning and electrospraying. Those mechanisms are important for the continuous and robust production and duplication of the EHDA products. Meanwhile, as more and more EHDA products are going into the commercial markets, these mechanisms for optimizing the production processes, and other related issues such as energy-saving, safety implementation, and environmental friendliness and projecting their places for the final social benefits and a better people life [71,72,73,74]. In this work, the mechanism for the strange phenomenon of many satellites is diagrammed in Figure 5. In the left part, a whole electrospraying process is sketched, i.e., a Taylor one, to a convergent point, and later to the Coulombic explosion region, in which the droplets were continuously splitted and reduced, until the formation and deposition of solid particles on the collector.

In the right part of Figure 5, two different fission processes are sketched. One kind was the uniform fission, by which the final electrosprayed particles were generated, as indicated in Figure 3a, where the surfaces of microparticles are smooth. The other kind was uneven fission, by which many satellites were formed during the electrospraying processes and were around the electrosprayed microparticles (Figure 3a). Their diameters were estimated to be several decades nanometers. In the electrospraying solution, the three solvents acetone, ethanol, and DMAc had their own main uses. CA is soluble in acetone and BH is soluble in DMAc. Although ethanol is a non-solvent for both BH and CA, it is useful for keeping the stretching state of CA molecules and, in turn, for promoting a stable and robust EHDA process. However, these solvents have different boiling points (56 °C, 78 °C, and 164 °C for acetone, ethanol, and DMAc, respectively) and different volatility. During the Coulombic explosion processes, it was possible that some droplets had more DMAc and kept a longer time period of the fluid state. Meanwhile, the surface charges may have different densities. Under these conditions, some sub-electrospraying processes may occur, by which the satellites are formed around the microparticles.

### 3.3. The Physical State and Compatibility

**Figure 6:**
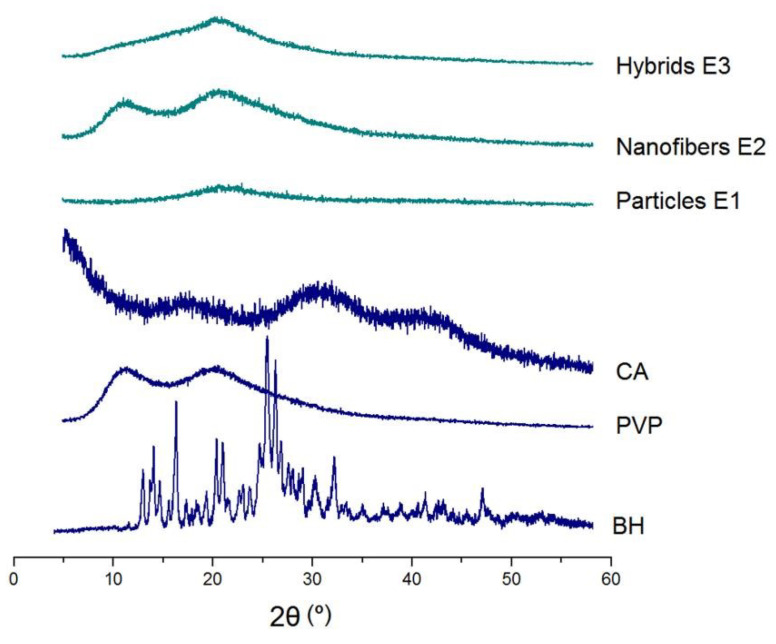
XRD patterns of the raw materials (CA, PVP, and BH) and their EHDA products (hybrids E3, nanofibers E2, and particles E1).

XRD patterns of the raw materials (CA, PVP, and BH) and their EHDA products (hybrids E3, nanofibers E2, and their combinations) are included in Figure 6. Just as anticipated, BH had many sharp Bragg peaks in its pattern due to the crystalline state of the original powders. In contrast, the hydrophilic polymer PVP K90 and insoluble polymer CA were all amorphous materials and, thus, only humped on their XRD patterns. The three EHDA products, i.e., the electrosprayed microparticles E1, the electrospun nanofibers E2, and the electrospun hybrids E3, showed no any sharp peaks in their patterns. These phenomena suggested that the drug BH was converted into amorphous composites in all of them after the electrospraying or electrospinning processes. An amorphous state of the drug is favorable for its dissolution and the manipulation of a certain controlled release profile from the matrices [75,76].

**Figure 7:**
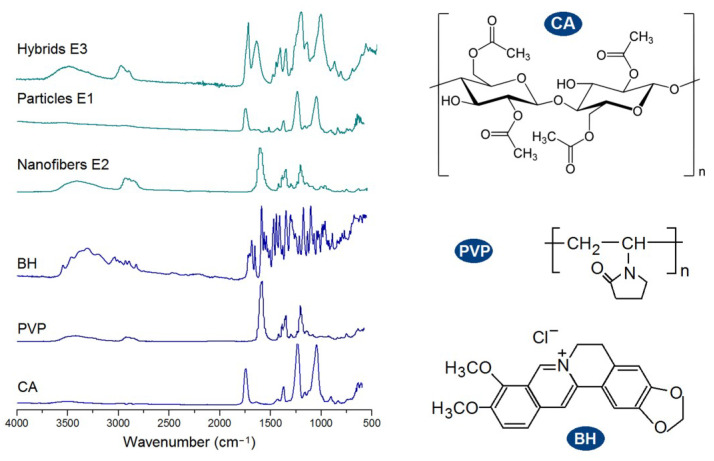
FTIR spectra of the raw materials (CA, PVP, and BH) and their EHDA products, and the molecular formats of the components within the EHDA products (CA, PVP, and BH).

FTIR spectra of the raw materials (CA, PVP, and BH) and their EHDA products are shown in the left part of Figure 7. The molecular formats of CA, PVP, and BH are shown in the right part of Figure 7. CA had its characteristic peaks at 1724, 1376, 1236, and 1051 cm^−1^. PVP had its characteristic peaks at 1662, 1423, and 1291 cm^−1^. BH had the typical absorbance of 1740, 1598, and 1504 cm^−1^, owing to the three benzene rings in one BH molecule. Compared with the raw materials, the spectra of microparticles E1 were almost the same as CA with little hints from BH, suggesting E1 particles were composites. Similarly, the spectra of electrospun nanofibers E2 were similar to PVP, giving a hint that BH formed composites with PVP. In spectra of E1 and E2, the substantial decrease and even disappearance in the intensities of characteristic peaks and peaks in the finger regions of BH should be attributed to the secondary interactions between the drug BH and the polymeric carriers. These secondary interactions include hydrogen bonding, hydrophobic interactions, and electrostatic interactions, which favor the compatibility between the drug and its carrier and are beneficial to the stability of formed binary composites [77,78]. Compared with spectra of E1 and E2, the hybrids E3′s spectra, on one hand, had also no BH sharp peaks and, thus, suggest an amorphous state of BH in them. On the other hand, E3′s spectra was a superimposition of the spectra of E1 and E2 to a certain extent, suggesting that the ternary EHDA products E3 were hybrids of the two binary composites.

### 3.4. In Vitro Drug Release Profiles

**Figure 8:**
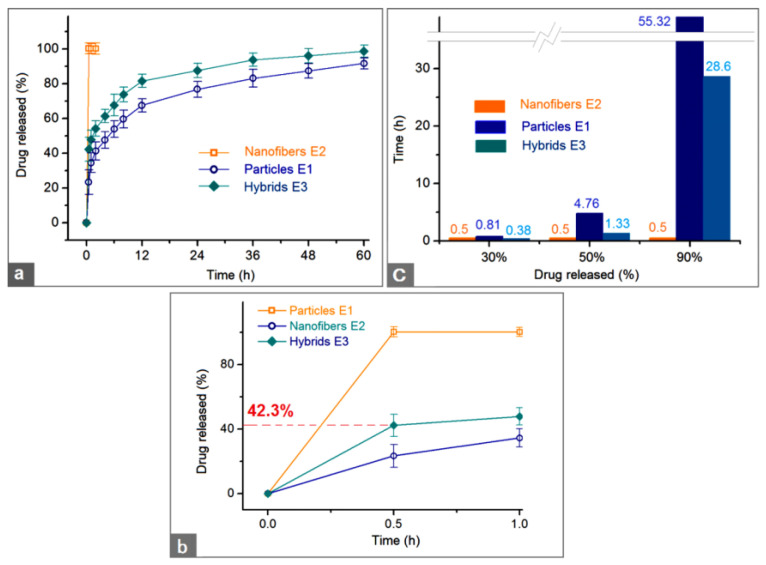
The in vitro dissolution test results: (**a**,**b**) drug accumulative release percentage (%) vs. sampling time point (h) for a whole experimental time and the first hour, respectively; and (**c**) estimated durations vs. a certain percentage of BH (30%, 50%, and 90%) that was released from the three EHDA products.

The pre-determined calibration equation for BH was *A* = 0.0688 × *C* − 0.0047 (R = 0.9999, and a linear range of 0.5 to 50 μg/mL), where *A* and *C* represent absorbance and BH concentration in μg/mL, respectively. The in vitro dissolution test results of the three types of EHDA products are shown in Figure 8. In Figure 8a,b, the curves were drawn according to the drug accumulative release percentage (%) vs. sampling time point (h), and in Figure 8c, the results are expressed according to the estimated durations vs. a certain percentage of BH (30%, 50%, and 90%).

The first pre-determined time point for sampling was 0.5 h after the samples were placed into the dissolution media. The electrospun nanofibers E2 released all the loaded BH through an erosion mechanism, i.e., the drug BH and the polymeric matrix PVP were co-dissolved into the dissolution media (Figure 8b). This pulsatile release can be attributed to the following three reasons besides the fine solubility of PVP in water: (1) the small diameter of the nanofibers and the related large surface area, (2) the amorphous state of the drug BH, (3) the 3-D web structure of the fibrous mats and the related high porosity [79]. Compared with a 100% percentage release in 0.5 and 1 h, the electrosprayed microparticles E2 and electrospun hybrids E3 released 23.4 ± 7.1% and 42.3 ± 6.8% after 0.5 h dissolution and 34.5 ± 5.6% and 47.9 ± 5.3% within one-hour dissolution, respectively. After a time period of 60 h dissolution, microparticles E2 and hybrids E3 released 91.7 ± 3.2% and 98.6 ± 3.5%, respectively. These data suggested that the hybrids E3 could provide a typical biphasic release profile with a release amount of 42.3% at the first phase in a pulsatile manner and 56.3% (98.6–42.3%) at the second phase in a sustained manner. It seems that the electrosprayed particles E1 also furnished a biphasic release, i.e., 34.5% and 57.2% (91.7–34.5%) at the first and second phases, respectively. However, the release contents at different phases from hybrids E3 were intentionally tailored in a relatively accurate manner, whereas the release contents at different phases from the microparticles E1 were random and often uncontrollable. Thus, in pharmaceutics, this case was regarded as an abnormal phenomenon to drug sustained release, i.e., initial burst effect.

The drug controlled release advantages of electrospun hybrids E3 over the electrosprayed microparticles E1 can be further projected from Figure 8b. For a 30% release of the loaded BH, 0.38 h and 0.81 h were needed for the electrospun hybrids E3 and electrosprayed microparticles E1, respectively. Meanwhile, for a 50% release of the loaded BH, 1.33 h and 4.76 h were needed for the electrospun hybrids E3 and electrosprayed microparticles E1, respectively. For quickly reaching a therapeutic blood drug concentration, the faster the dosage forms can provide, the better effectiveness and compliance the patients have. From this standpoint, the hybrids E3 were apparently better than the microparticles E1.

For a 90% release of the loaded BH, 28.6 h and 55.32 h were needed for the electrospun hybrids E3 and electrosprayed microparticles E1, respectively. In drug sustained release, an abnormal phenomenon is a tailing-off release, in which the drug is exhausted very slowly from its carrier and cannot keep an effective therapeutic blood drug concentration. The release percentage between 90% and 100% often falls within this abnormal region and should be avoided. From this standpoint, hybrids E3 are better than microparticle E1 due to a smaller tailing-off release and also a terminal release amount (98.7 ± 3.5% and 91.8 ± 3.2% after 60 h for E3 and E1, respectively).

Additionally, the suspension fluid had an amount of 15.0 g microparticles E1 from electrospraying and 200 mL Fluid 2. The BH weight in the microparticles was 15.0 × 20% = 3.0 g. The BH weight in the nanofibers was 4.0 × (200 mL/400 mL) = 2.0 g. Thus, in theory, the drug released in the first phase should be 2.0/(2.0 + 3.0) × 100% = 40%. The released BH from hybrids E3 after 0.5 and 1 h were 42.3 ± 6.8% and 47.9 ± 5.3%, respectively. The values were larger than the theoretically calculated value of 40%. This case should be attributed to both the preparation of working suspensions for creating hybrids E3 and drug release from the particles. Some surface BH on the surface of electrosprayed microparticles E1 should re-dissolve into the suspensions, making a little higher drug concentration in the hydrophilic PVP. Further studies may be designed to improve the accuracy of drug release contents at different phases, e.g., a blank CA coating on the microparticles through coaxial electrospraying.

### 3.5. Drug Release Mechanism

**Figure 9:**
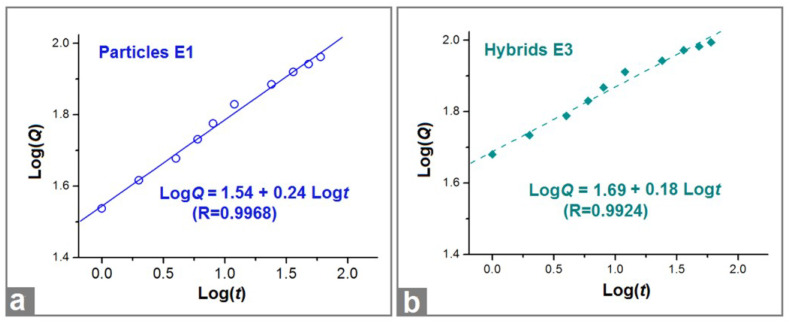
The regressed equations drawn from the in vitro BH release data of particles E1 (**a**) and hybrids E3 (**b**).

To disclose the drug release mechanism, the Peppas equation (Q = kt^n^, where Q is the drug release content, k is a constant, and t is an indicator of drug release behaviors [80]) was exploited to regress the BH release data achieved during the in vitro dissolution tests (time ≥ 1 h). The results for the electrosprayed microparticles E1 and electrospun hybrids E3 are exhibited in Figure 9. For particles E1, the regressed equation was Log*Q* = 1.54 + 0.24 Log*t* (R = 0.9968). For the second phase of hybrids E3, the regressed equation was Log*Q* = 1.69 + 0.18 Log*t* (R = 0.9924). Both EHDA products had an n value smaller than the critical judgment value of 0.45, suggesting that BH was released from the CA microparticles through a typical Fickian diffusion mechanism, regardless of a sole state of microparticles E1 or a co-existing state with hydrophilic polymer PVP in hybrids E3.

**Figure 10:**
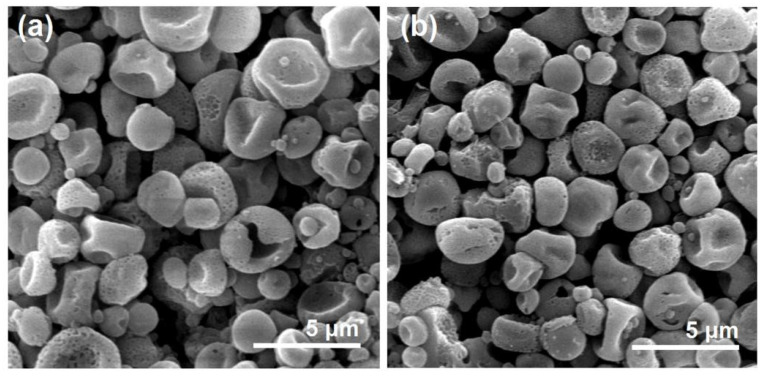
The SEM images of the residue microparticles after the exhaustion of BH molecules from the microparticles E1 (**a**) and the hybrids E3 (**b**).

After a full time period of 60 h dissolution, the residue particles were taken out from the in vitro dissolution vessels and naturally dried. These particles experienced SEM evaluations. Their images are exhibited in Figure 10. Compared with the previous images before dissolution, both microparticles E1 (Figure 10a) particles in hybrids E3 (Figure 10b) lost their original smooth surface and solid state, but they exhibited a porous surface and a more concave morphology. Apparently, those surface holes and deformed surface morphology (resulted from a void inner) were direct outcomes owing to the removement of loaded BH molecules, providing an intuitive clue of a drug diffusion mechanism from the insoluble CA matrix. Compared with previous reports of biphasic release from electrospun core-sheath nanofibers [81], hybrids of electrospun nanofibers and casting films [82], and electrospun Janus nanofibers [83], the present trans-scale hybrids E3 showed a longer sustained release second phase and a better biphasic release profile.

**Figure 11:**
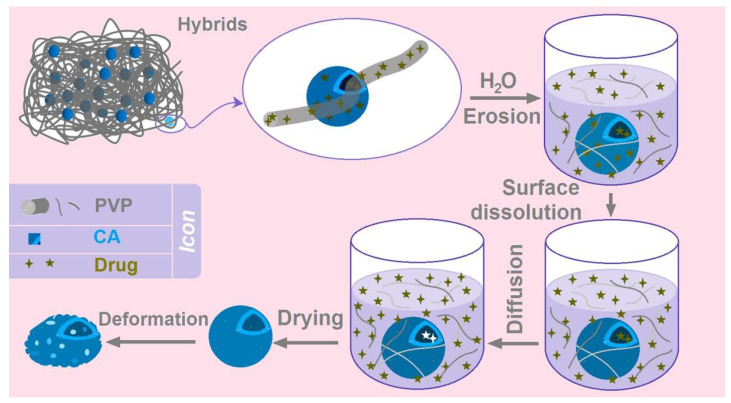
The mechanism about the biphasic release of BH from the hybrids of electrospun PVP nanofibers and electrosprayed CA microparticles.

Based on the above-mentioned analyses, a diagram is shown in Figure 11. The combined mechanism of the BH biphasic release from the hybrids E3 containing electrospun PVP nanofibers and electrosprayed CA microparticles was clear. When the hybrids E3 are placed into the dissolution media, the BH-PVP nanofibers will be rapidly dissolved. This is a typical erosion process for the fast release of BH in the first phase. Later, the BH molecules distributed or absorbed on the surface of CA microparticles would dissolve into the dissolution media, by which the routes for water molecules’ penetration into the inner sections of CA particles gradually open. Along with the penetration of water molecules from surface to the core of CA microparticles, the loaded drug BH molecules would be free and inversely diffused from the CA particles to the bulk solution. During all the processes, the CA skeleton is insoluble and keeps the routes for diffusions and exchanges of both water and BH molecules. In theory, the diffusion process will not be terminated until a uniform BH distribution all over the bulk solution and a dynamic absorbance balance between the dissolution media and solid CA skeleton. After the CA skeletons are fetched out and dried, it is inevitable for them to experience some deformations.

New methods for human health are always highly desired [84,85,86,87,88,89]. Today, on one hand, numerous strategies were reported in the literature to create novel functional ingredients on a molecular scale with chemical reactions as the fundamental supports [90,91,92,93,94,95]. On the other hand, new methods bloomed in manipulating the molecules into nano aggregates from both “top-down” manner and “bottom-up” way [96,97,98,99,100,101]. In this study, a new concept was demonstrated and a trans-dimensional strategy was explored to generate functional hybrid materials through a combination of nanoproducts and products at microscale for an improved final functional performance. Based on the protocols reported here, there are a wide variety of possibilities for conceiving novel functional materials in the future.

## 4. Conclusions

In this study, a sequential EHDA process was successfully developed for creating a new kind of medicated hybrids E3. The hybrids E3 contained both BH-loaded hydrophilic PVP nanofibers and insoluble BH-loaded CA microparticles. The key element was that the electrosprayed BH-CA microparticles were insoluble in the solvent mixture of DCM and DMAc (with a volume ratio of 9:1) and, thus, an electrospinnable suspension was prepared, and in turn, the nanofiber-microparticle hybrids E3 were achieved through the single-fluid electrospinning process. The routine characterization results indicated that the hybrids E3 were a mixture of particles and nanofibers with BH distributed in the PVP and CA matrices in an amorphous state. In vitro dissolution tests demonstrated that the hybrids E3 were able to furnish the designed biphasic release profile, with a 42.3% drug release at the first immediate release phase and a 56.3% drug release at the second phase in a sustained manner. The BH molecule release was manipulated through a combination of molecular erosion mechanism and the typical molecular Fickian diffusion mechanism. This research paves a new way for developing functional materials through organizing materials at different scale levels and with different outer shapes.

## Figures and Tables

**Table 1:** Parameters for the EHDA processes.

No.	EHDA Process	Working Fluid	Experimental Conditions			Drug Contents	Morpho- Logy
			V (kV)	F (mL/h)	D (cm)		
E1	Electrospraying	Fluid 1 ^a^	20	1.0	20	20.0%	Particles
E2	Electrospinning	Fluid 2 ^b^	8	2.0	20	10.0%	Fibers
E3	Sequential EHDA process	Fluid 3 ^c^	12	2.0	20	14.3%	Hybrids

^a^ Fluid 1: An amount of 5.0 g BH and 20.0 g CA were co-dissolved in 400 mL of the solvent mixture comprising acetone/ethanol/DMAc with a volume ratio of 4:1:1. ^b^ Fluid 2: An amount of 36.0 g PVP and 4.0 g BH were co-dissolved into 400 mL mixture of DCM and DMAc with a volume ratio of 9:1. ^c^ Fluid 3: An amount of 15.0 g microparticles E1 from electrospraying were suspended into 200 mL Fluid 2 uniformly through continuous stirring.

## Data Availability

The data supporting the findings of this manuscript are available from the corresponding authors upon reasonable.
